# Cognitive aspects of motor control deteriorate while off treatment following subthalamic nucleus deep brain stimulation surgery in Parkinson’s disease

**DOI:** 10.3389/fneur.2024.1463970

**Published:** 2024-12-18

**Authors:** Miranda J. Munoz, Rishabh Arora, Yessenia M. Rivera, Quentin H. Drane, Gian D. Pal, Leo Verhagen Metman, Sepehr B. Sani, Joshua M. Rosenow, Lisa C. Goelz, Daniel M. Corcos, Fabian J. David

**Affiliations:** ^1^Department of Physical Therapy and Human Movement Sciences, Northwestern University, Chicago, IL, United States; ^2^USF Health Morsani College of Medicine, University of South Florida, Tampa, FL, United States; ^3^Department of Medical Social Sciences, Northwestern University Feinberg School of Medicine, Chicago, IL, United States; ^4^Creighton University School of Medicine, Creighton University, Omaha, NE, United States; ^5^Department of Neurology, Rutgers-Robert Wood Johnson Medical School, New Brunswick, NJ, United States; ^6^Department of Neurology, Northwestern University Feinberg School of Medicine, Chicago, IL, United States; ^7^Department of Neurosurgery, Rush University Medical Center, Chicago, IL, United States; ^8^Department of Neurological Surgery, Northwestern University Feinberg School of Medicine, Chicago, IL, United States; ^9^Department of Kinesiology and Nutrition, University of Illinois at Chicago, Chicago, IL, United States

**Keywords:** Parkinson’s disease, deep brain stimulation surgery, saccade, latency, reach, reaction time, cognitive aspects

## Abstract

**Introduction:**

The long-term effects of surgery for subthalamic nucleus deep brain stimulation (STN-DBS) on cognitive aspects of motor control for people with Parkinson’s disease (PD) are largely unknown. We compared saccade latency and reach reaction time (RT) pre- and post-surgery while participants with PD were off-treatment.

**Methods:**

In this preliminary study, we assessed people with PD approximately 1 month pre-surgery while OFF medication (OFF-MEDS) and about 8 months post-surgery while OFF medication and STN-DBS treatment (OFF-MEDS/OFF-DBS). We examined saccade latency and reach reaction time (RT) performance during a visually-guided reaching task requiring participants to look at and reach toward a visual target.

**Results:**

We found that both saccade latency and reach RT significantly increased post-surgery compared to pre-surgery. In addition, there was no significant change in Movement Disorder Society-Unified Parkinson’s Disease Rating Scale (MDS-UPDRS) Part III score.

**Discussion:**

We found detrimental post-surgical changes to saccade latency and reach RT. We discuss the potential contributions of long-term tissue changes and withdrawal from STN-DBS on this detrimental cognitive effect.

## 1 Introduction

Following surgery for subthalamic nucleus deep brain stimulation (STN-DBS), people with Parkinson’s disease (PD) frequently experience a beneficial acute microlesion effect (1–11). A microlesion occurs after electrode insertion, resulting from damage to the cells within the penetrated nucleus. The acute microlesion effect is an improvement in PD motor signs, typically measured as a decrease in OFF treatment Unified Parkinson’s Disease Rating Scale (UPDRS) Part III score. However, the beneficial acute microlesion effect on PD motor signs declines in the months following surgery (1, 7–9).

Much less is known about the effects of electrode insertion on the cognitive aspects of movement, such as latency and reaction time (RT), which both reflect attention and processing speed (12–14). Interestingly, Antoniades et al. (15) found significantly increased or worsened visually-guided saccade latency 24 h post-surgery compared to pre-surgery, even while UPDRS Part III scores were improved in the same participants. While Antoniades et al. (15) showed that the detrimental effect on saccade latency declined after 3–4 weeks while ON medication before stimulation initiation, it is unknown how saccade latency is affected while OFF treatment after months post-surgery. Evaluation of the long-term effects post-surgery is important to determine if the acute detrimental effects persist. In this preliminary study, we aimed to determine if there were detrimental long-term pre- to post-surgical changes of both saccade latency and reach RT while participants were OFF treatment. While no studies have reported post-surgical changes in RT of a reach, it is important to determine if the effect of surgery on cognitive aspects of movement can be identified across effectors. A small cohort of people with PD performed a visually-guided reaching task about 1 month pre-surgery while OFF medication (OFF-MEDS) and about 8 months post-surgery while OFF medication and STN-DBS treatment (OFF-MEDS/OFF-DBS). We examined the change in saccade latency and reach RT post-surgery compared to pre-surgery.

## 2 Methods

Northwestern University and Rush University Medical Center Institutional Review Boards approved this study, and all experiments were completed in accord with the Helsinki Declaration of 1975. We obtained informed consent from all participants. Nine participants with PD completed OFF-MEDS testing about 1 month before undergoing STN-DBS surgery and OFF-MEDS/OFF-DBS testing on average 8 months after surgery (range = 6–10 months, average = 7.89 months) (Table 1). All participants with PD had a good response to bilateral STN-DBS, as evidenced by an average of 66% decrease in OFF-MEDS/ON-DBS Movement Disorder Society-UPDRS (MDS-UPDRS) Part III score compared to OFF MEDS/OFF-DBS.

**Table 1:** Demographics and clinical scores.

ID	Age (years)	Disease duration (years)	Time since surgery (months)	MDS-UPDRS III			MoCA		LEDD	
				PRE	POST	ON	PRE	POST	PRE	POST
1	60	16	6	42	43	12	30	30	1940	960
2	65	7	8	48	61	10	29	27	600	450
3	68	4	9	39	34	14	27	27	600	0
4	63	5	10	54	44	21	24	25	600	350
5	72	13	6	52	70	14	27	27	2,740	400
6	66	4	8	30	28	15	29	28	570	285
7	69	18	8	52	59	25	28	27	1,350	500
8	65	9	7	54	62	19	27	30	380	0
9	66	12	9	63	68	31	28	23	1,435	200
Mean	66.00	9.78	7.89	48.22	52.11	17.89	27.67	27.11	1135.00	349.44
SD	3.46	5.24	1.36	9.81	15.23	6.79	1.73	2.20	799.08	291.30

LEDD, Levodopa Equivalent Daily Dose; MDS-UPDRS III, Movement Disorder Society-Unified Parkinson’s Disease Rating Scale; MoCA, Montreal Cognitive Assessment; ON, post-surgery (OFF-MEDS/ON-STIM); PRE, pre-surgery (OFF-MEDS); POST, post-surgery (OFF-MEDS/OFF-STIM); SD, standard deviation.

**Figure 1:**
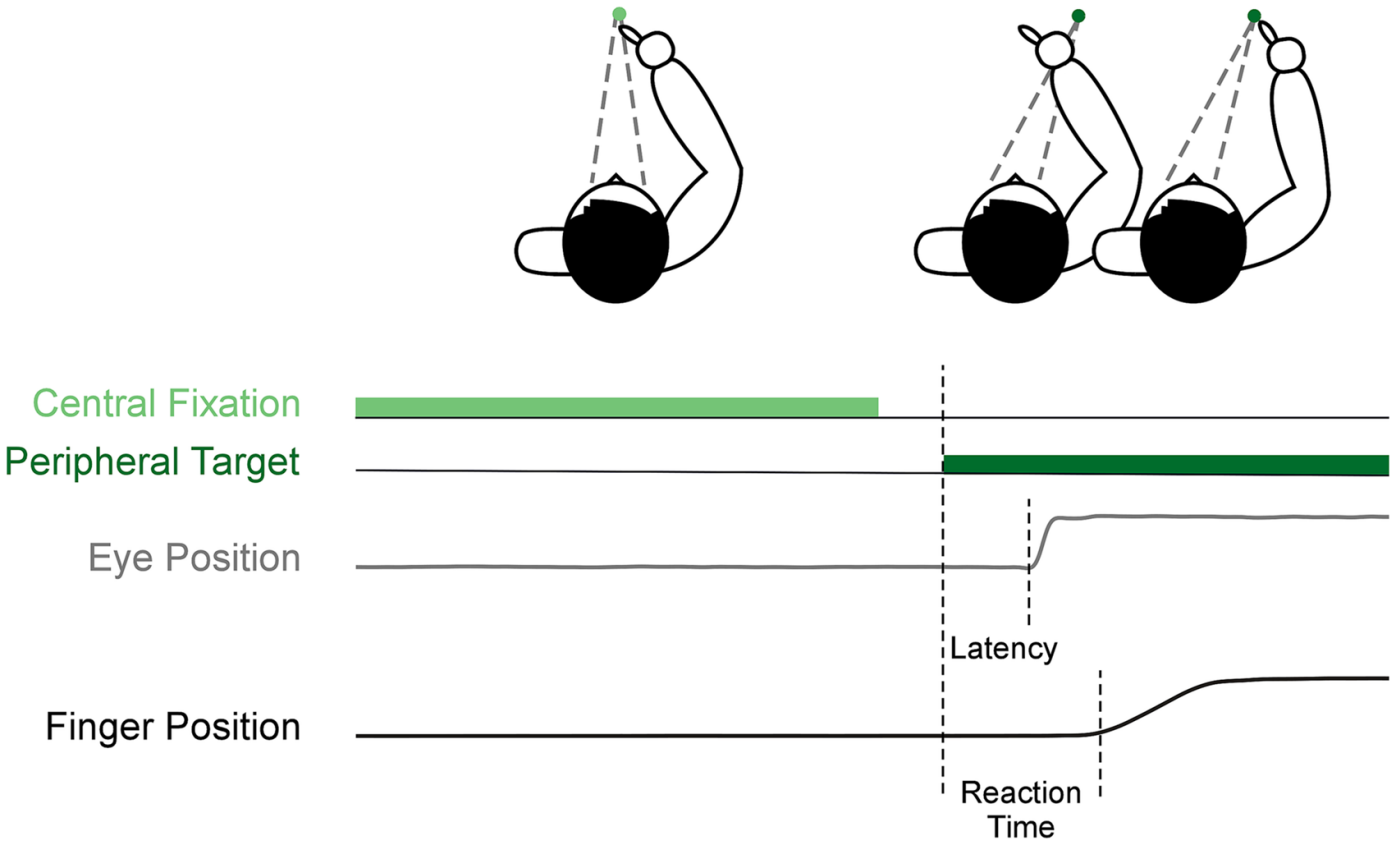
Visually-guided reach task. Each trial began with central fixation (light green), then after a 200 ms gap, a peripheral target appeared to the right (dark green). This was the cue for participants to saccade and reach to the target as shown by the example eye position (gray) and finger position (black).

A detailed description of inclusion criteria, data collection, data processing, and statistics can be found in our previous publication (16). In brief, all antiparkinson medications were withdrawn overnight for at least 12-h (17), and bilateral STN-DBS was washed-out for at least 3-h before testing (18). We collected antiparkinson medication information to calculate the levodopa equivalent daily dose (LEDD), administered the Montreal Cognitive Assessment (MoCA) while participants were ON-MEDS or ON-MEDS/ON-DBS, and administered the MDS-UPDRS Part III while participants were OFF-MEDS or OFF-MEDS/OFF-DBS. The MoCA was completed ON treatment because it was administered on a separate intake day. We compared pre- to post-surgical averages in these measures using two-tailed paired t-tests. Additionally, we recorded eye movements (Eyelink II, SR Research Ltd) and upper limb movements (Optotrak 3020, Northern Digital) during a visually-guided reaching task. The task involved participants fixating on a central cue (2000–3000 ms) and then looking at and reaching to a peripheral rightward target after a 200 ms gap (Figure 1). We calculated our primary outcome measures, saccade latency and reach RT, as the time difference between target onset and movement onset of the eyes and upper limb, respectively, as identified by a custom MATLAB code (The MathWorks Inc).

Pre- to post-surgical differences were assessed using linear mixed-effect regression models with the fixed effect being time (pre vs. post) and the random effect being participant to account for the dependence between trials within participant. Latency and RT were log transformed because the data was right skewed, and we present the estimated difference transformed back to the original scale (Est diff_BT_). Back-transformation was performed on the pre- and post-surgery estimated means and then the difference in means was calculated. To determine the influence of change in disease severity on latency and RT, change in MDS-UPDRS Part III was included as a covariate. However, this change score had no significant effect and did not improve the model. Therefore, it was not included in the final models presented.

## 3 Results

**Figure 2:**
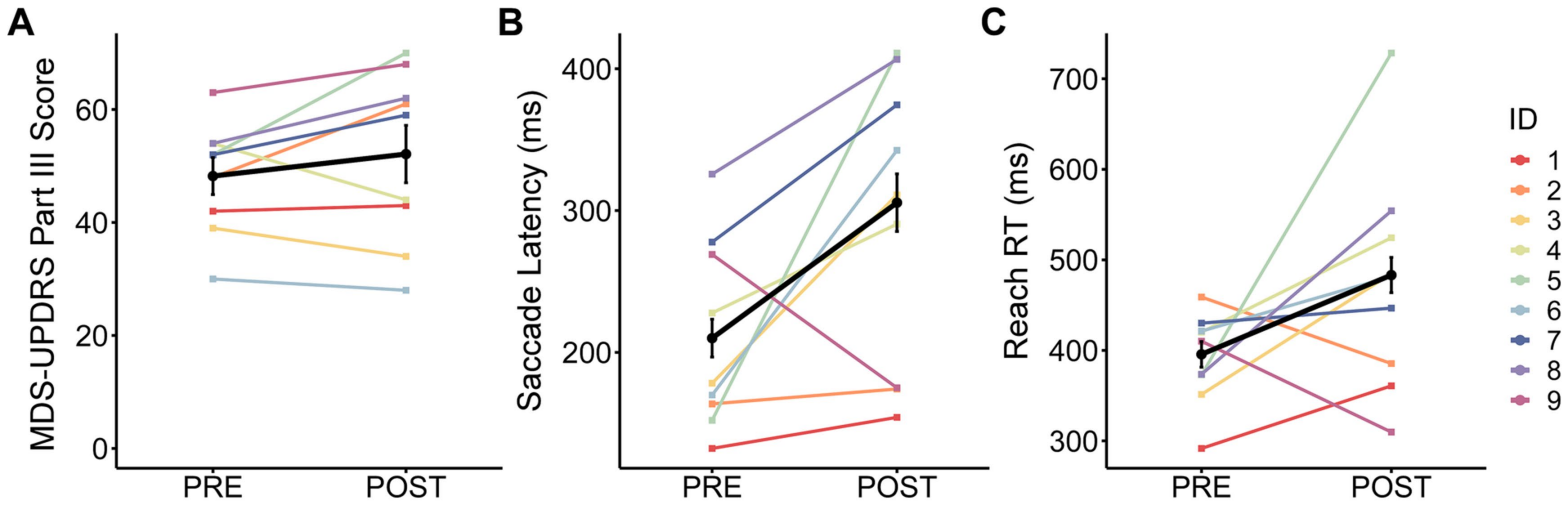
Change from pre- to post-surgery. The observed change in group mean ± standard error (black line and error bars) and individual means (color lines for each ID) from pre- to post-surgery for **(A)** Movement Disorder Society-Unified Parkinson’s Disease Rating Scale (MDS-UPDRS) Part III score, **(B)** saccade latency, and **(C)** reach reaction time (RT).

MDS-UPDRS Part III scores were not significantly different between post-surgery (OFF-MEDS/OFF-DBS) and pre-surgery (OFF-MEDS) (Figure 2A; Table 2). Similarly, MoCA scores were not significantly different between post-surgery (ON-MEDS/ON-DBS) and pre-surgery (ON-MEDS) (Table 2). Finally, levodopa equivalent daily dose was significantly decreased by 69% post-surgery compared to pre-surgery (Table 2).

**Table 2:** Summary of statistics describing the change from pre- to post-surgery.

Changes in disease severity, cognition, and medication dosage^a^					
Outcome	Est Diff	95% CI	*t*	df	*p*
MDS-UPDRS Part III	−3.89	−10.68 to 2.91	−1.32	8	0.223
MoCA	0.56	−1.13 to 2.24	0.76	8	0.468
LEDD (mg)	785.56	255.76 to 1315.36	3.42	8	0.009

Changes in saccade latency and reach RT^b^						
Outcome	Est Diff_BT_	Est Diff	95% CI	*F*	df	*p*
Saccade latency (ms)	72.15	0.32	0.18 to 0.46	20.09	121	< 0.001
Reach RT (ms)	76.72	0.18	0.09 to 0.28	13.64	121	< 0.001

^aResults from paired t-tests including the mean difference, 95% confidence interval, t-value, degrees of freedom, and p-value. bResults from linear mixed models including the estimated difference back-transformed, estimated difference in log scale, 95% confidence interval, F-value, degrees of freedom, and p-value. Outcomes were determined from 131 total trials.^

Saccade latency was significantly increased by 38% post-surgery (OFF-MEDS/OFF-DBS) compared to pre-surgery (OFF-MEDS) (Est diff_BT_ = 72.15 ms; Figure 2B; Table 2). Additionally, reach RT was significantly increased by 20% post-surgery (OFF-MEDS/OFF-DBS) compared to pre-surgery (OFF-MEDS) (Est diff_BT_ = 76.72 ms; Figure 2C; Table 2).

## 4 Discussion

We found significant detrimental effects on the cognitive aspects of movement, i.e., saccade latency and reach RT, when participants were tested 8 months post-surgery compared to pre-surgery. Not only were these effects statistically significant, but also the magnitude of the change is much larger than our previously reported effects of medication and STN-DBS on these same measures (16). Meanwhile, there was no difference between pre- and post-surgical OFF treatment MDS-UPDRS Part III scores or ON treatment MoCA scores and an expected decrease in post-surgical LEDD. We will discuss (1) four potential factors that could impact the post-surgical change in saccade latency and reach RT, (2) the lack of change in our clinical scores, and (3) how these results compare to the effect of stimulation.

### 4.1 Long-term tissue changes due to implantation

One potential factor is the long-term tissue changes due to lead implantation. While the acute microlesion in the STN benefits motor performance, the lead could also impact neural function in the tissue it passes through to impair cognitive performance. On a cellular level, animal and human studies have shown long-term tissue changes due to implantation, including sustained gliosis around the implant (19). This long-term gliosis can alter the neuronal function in the vicinity of the implant, the connectivity within associated circuits, and the volume and metabolic activity of connected regions (19). These tissue changes can impact many different brain areas. At our surgical centers, the lead typically passes through the middle frontal gyrus, frontal white matter, reticular thalamus, internal capsule, thalamic fasciculus and lenticular fasciculus, zona incerta, and STN. Several studies have hypothesized that the electrode trajectory through frontal regions could negatively affect cognitive functions (20, 21). Similarly, studies have shown that the location of the electrode cortical entry point is related to post-surgical verbal fluency decline (22, 23). Changes to frontal activity could impact saccade latency and reach RT directly or through its connections with the parietal cortex, which is associated with attention and sensory-motor transformations (24, 25). Additionally, the lead implantation could intersect and damage white matter tracts that carry motor and oculomotor information (26), although the exact tracts that are damaged are still being explored (27). For example, the internal capsule contains projections leading to the superior colliculus that can affect saccade latency (28) and the longitudinal fasciculus contains projections associated with spatial coordination and attention, which can impact both saccade and reach performance (29). Finally, damage to the zona incerta could also impact saccade latency because it sends projections to the superior colliculus and receives input from frontal and parietal eye fields (30). We have previously shown that turning on STN-DBS decreases saccade latency compared to OFF-DBS in this same task (16). However, STN-DBS does not significantly improve reach RT (16), suggesting that long-term tissue changes could still affect performance even with stimulation.

### 4.2 Electrophysiological changes after withdrawal of chronic STN-DBS

Another potential factor is the electrophysiological neural change after withdrawal of chronic STN-DBS. For instance, increased alpha band power has been shown after 1-h withdrawal of chronic STN-DBS at 3 years compared to initial programming (31). Alpha power has been related to attention (31) and increased alpha power could contribute to or reflect greater attentional deficits, leading to increased saccade latency and reach RT. There is also some evidence that chronic stimulation could induce changes in brain volume (32, 33) and functional network organization (34), but no conclusions can yet be drawn (35). Network changes due to chronic STN-DBS could explain the difference between our findings and those of Antoniades et al. (15). While Antoniades et al. (15) reported a return to pre-surgery saccade latency values within 3–4 weeks before stimulation was initiated, we show a substantial prolongation of latency at 6–10 months after withdrawal from chronic STN-DBS.

### 4.3 Reduction in medication

Another potential factor is the reduction in medication post-surgery due to STN-DBS. One hallmark benefit of STN-DBS is that daily medication dosage can be reduced (36). We demonstrated that LEDD was significantly reduced post-surgery, such that medication dosage was reduced, on average, by 69% in our sample. This reduction indicates that our participants had a beneficial and typical response to STN-DBS itself.

If the reduction of medication was an important underlying factor, we would expect that reducing medication would result in a decrease in latency. This is because we have previously reported that medication prolongs saccade latency compared to being OFF medication (16, 37). However, we observe the opposite relationship: a post-surgical reduction in medication accompanied by a prolongation of saccade latency. Thus, it is unlikely that the post-surgical reduction in medication dosage accounts for our findings.

### 4.4 Disease progression

Finally, another potential factor is disease progression. In PD, disease progression is typically characterized by a change in UPDRS Part III score. While disease progression is not linear and changes with disease stage, a 2.4 point increase is expected per year on average early in the disease (38). In our sample, change in MDS-UPDRS Part III score was not a significant covariate and, when included in our linear mixed model, had no effect on the significance of the pre- to post-surgical differences in saccade latency or reach RT. This suggests that disease progression did not significantly affect our results.

The effect of disease progression on visually-guided saccades or reaching has not been studied. However, a preliminary study reported that disease progression over 12 months did not change antisaccade latency in people with PD without STN-DBS (39). Therefore, it is unlikely that the observed increase in latency and RT is solely due to disease progression considering the magnitude of change.

### 4.5 Lack of change in MDS-UPDRS III and MoCA scores

We found that there was no change in OFF treatment MDS-UPDRS Part III or ON treatment MoCA scores post- vs. pre-surgery, demonstrating that our saccade and reach findings cannot be explained by a global deterioration of motor or cognitive performance. Although it should be noted that since MoCA scores were collected ON treatment, it is possible that deterioration of general cognitive performance could be detected OFF treatment and this should be considered in future studies. Previous reports on UPDRS Part III similarly demonstrated that there was no change between pre- and 1–48 months post-surgery (7, 9, 40). In addition, recent studies have shown that there was no change in MoCA score between pre- and 6–12 months post-surgery (41, 42). Thus, our data agrees with previous literature that there are limited changes in average motor severity or general cognitive function in the months post-surgery. This signifies the importance of using sensitive and quantitative behavioral measures, such as saccade latency and reach RT, to elucidate the behavioral changes after surgery.

### 4.6 Comparison with the beneficial effects of STN-DBS

Previous studies reported that bilateral STN-DBS decreased saccade latency in a saccade only task (43–45) and decreased reach RT in a simple RT task (45–47) compared to OFF stimulation. Similarly, our previous study using the same visually-guided reach task found that bilateral STN-DBS significantly decreased saccade latency, but only non-significantly decreased reach RT compared to OFF stimulation (16). This is important to note because STN-DBS reduced latency and RT toward healthy control levels. Therefore, while our current findings show an OFF treatment post-surgical detriment to saccade latency and reach RT, turning on STN-DBS will likely counteract this detriment. Although it should be considered that long-term tissue changes could still affect performance even with stimulation, as bilateral STN-DBS did not significantly improve reach RT in our previous study (16).

As individuals with STN-DBS are typically being stimulated, the detriment we present here should not impact daily function. However, our results indicate that there are significant behavioral changes due to implantation itself, withdrawal of chronic stimulation, or both, suggesting that long-term neurological changes are occurring. These long-term changes are important to understand in the instance that a patient discontinues STN-DBS temporarily or permanently, or that STN-DBS efficacy declines. Understanding the OFF treatment behavioral changes also highlights which circuits are being affected by implantation and stimulation.

### 4.7 Limitations

There are several limitations of this preliminary study. First, we had a small sample size with data from only 9 individuals. However, previous studies have used a similar sample size to demonstrate the difference between pre- and post-surgery (10, 15, 48). Second, there is a high variability between participants in task performance. However, the majority of individuals have increased saccade latency (8 out of 9) and reach RT (7 out of 9) post-surgery, so the direction of change was relatively consistent in our sample. Third, there is high variability in our participant demographics and clinical scores, but this is representative of the natural variability of those individuals who opt for STN-DBS. While there was a high variability in individual change in MDS-UPDRS Part III, this variability has been reflected in previous studies who have compared UPDRS Part III pre- and months post-surgery (7, 9, 40). We also confirmed that every participant had a beneficial response to bilateral STN-DBS, as shown by a decrease in MDS-UPDRS Part III score when ON stimulation (Table 1). Each of these limitations emphasizes the need for these findings to be replicated in a larger sample.

## 5 Conclusion

Our preliminary findings suggest that there are large increases in saccade latency and reach RT after STN-DBS surgery while OFF-MEDS/OFF-DBS compared to pre-surgery when people with PD are OFF-MEDS. We suggest that this detrimental effect on a cognitive aspect of movement could be due to long-term tissue changes after lead implantation and/or neural changes after the withdrawal of chronic STN-DBS.

## Data Availability

The raw data supporting the conclusions of this article will be made available by the authors, without undue reservation.
